# Investigating the effectiveness of an intraoperative decision support guided fluid therapy intervention on postoperative outcome of high-risk patients undergoing high-risk abdominal surgery: protocol for an international multicentre stepped-wedge cluster-randomised implementation trial

**DOI:** 10.1016/j.bjao.2025.100421

**Published:** 2025-06-05

**Authors:** Sean Coeckelenbergh, Amelie Delaporte, Damien Rousseleau, Jacques De Montblanc, Stephanie Roullet, Joanna Ramadan, Bernard Cholley, Alexandre Sitbon, Emmanuel Weiss, Maria-Christina Kassab, Sylvain Diop, Elsa Manzi, Marco Pustetto, Guillaume Porta Bonette, Pierre Gregoire Guinot, Philippe Guerci, Domien Vanhonacker, Francois Martin Carrier, Brenton Alexander, Joseph Rinehart, David Boldt, Tristan Grogan, Maxime Cannesson, Jacques Duranteau, Lamiae Grimaldi, Bruno Pereira, Alexandre Joosten

**Affiliations:** 1 Department of Anesthesiology & Perioperative Care, University of California, Irvine, Irvine, CA, USA; 2 Outcomes Research Consortium, Cleveland, OH, USA; 3 Department of Anesthesiology & Perioperative Medicine, University of California, Los Angeles, Los Angeles, CA, USA; 4 Department of Anesthesiology and Intensive Care Department, Claude Huriez Hospital, Lille University Hospital, Lille, France; 5 Department of Anesthesiology and Intensive Care, Bicêtre Hospital, Le Kremlin Bicêtre, France; 6 Department of Anesthesiology and Intensive Care, Paris-Saclay University, Paul Brousse Hospital, Villejuif, France; 7 Department of Anesthesiology, Intensive Care Perioperative Medicine, Hôpital Européen Georges Pompidou, Paris Cité University, Paris, France; 8 Department of Anesthesiology and Critical Care, La Pitié-Salpêtrière Hospital, Sorbonne University, Paris, France; 9 Department of Anesthesiology and Critical Care, Beaujon Hospital, Paris Cité University, Paris, France; 10 Department of Anesthesiology, Marie Lannelongue Hospital, Le Plessis-Robinson, France; 11 Department of Anesthesiology, Institut Mutualiste Montsouris, Paris, France; 12 Department of Anesthesia and Intensive Care, Grenoble-Alpes University Hospital, Grenoble, France; 13 Department of Anesthesiology and Perioperative Medicine, Pierre-Paul Riquet Hospital, Toulouse, France; 14 Department of Anesthesiology and Critical Care Medicine, Dijon University Medical Centre, University of Burgundy and Franche-Comté, Dijon, France; 15 Department of Anesthesiology, Nancy University Hospital, Nancy, France; 16 Department of Anesthesiology, Perioperative and Pain Medicine, Vrije Universiteit Brussel, Universitair Ziekenhuis Brussel, Brussels, Belgium; 17 Department of Anesthesiology, Montreal University Hospital, Montreal, QC, Canada; 18 Department of Anesthesiology, University of California, San Diego, La Jolla, CA, USA; 19 Department of Medicine Statistics Core, David Geffen School of Medicine, University of California, Los Angeles, Los Angeles, CA, USA; 20 Clinical Research Unit, Paris-Saclay University, Bicêtre Hospital, Le Kremlin Bicêtre, France; 21 Clinical Research Unit, Clermont-Ferrand University Hospital, Clermont-Ferrand, France

**Keywords:** acute kidney injury, complications, fluid therapy, goal directed therapy, haemodynamic monitoring, haemodynamic optimisation, high-risk surgery, mortality, outcome, surgery

## Abstract

**Background:**

Inappropriate fluid administration can impact patient outcome. Intraoperative advanced haemodynamic monitoring coupled with a treatment protocol based on stroke volume optimisation can help determine the appropriate timing for fluid boluses. Although recommended by several anaesthesia societies, this strategy is rarely implemented because protocols are complex and compliance is often poor. The Acumen Assisted Fluid Management (AFM) software is a decision support system that uses machine learning to predict fluid responsiveness and individualise fluid therapy. AFM reportedly predicts fluid responsiveness better than clinicians, decreases preload-dependent states, and improves both macro- and microcirculatory variables. The goal of this international multicentre stepped-wedge cluster randomised trial is to test whether implementing AFM during high-risk surgery improves patient outcome.

**Methods:**

The trial is ongoing in 16 academic hospitals in France, Belgium, Canada, and the USA. All centres (clusters) deliver routine care (control arm) at the start of the study and crossed over (one way) to AFM-guided fluid therapy (intervention arm). The time when different centres switch to AFM is randomised by an independent statistician. At the end of the trial, all centres will cross over to the intervention group. The primary outcome is a composite of major complications and death 30 days after surgery that will be analysed as intention-to-treat. A total of 2000 patients are required to detect a relative 20% differences in the primary outcome between groups.

**Conclusions:**

This trial is powered to provide evidence on whether implementing AFM is effective in reducing postoperative complications in high-risk patients after high-risk abdominal surgery.

**Clinical trial registration:**

NCT06011187.

## Introduction

Among the 313 million major noncardiac surgical procedures performed each year throughout the world, high-risk surgeries account for nearly 80% of perioperative deaths.^1^ Because the underlying cause of death in patients is frequently tissue hypoxemia, maintaining adequate intraoperative cardiac output (CO) and oxygen delivery during surgery may prevent damage to vital organs and decrease morbidity and mortality. Goal-directed stroke volume (SV) optimisation with fluids may improve outcomes in high-risk surgical patients^2^,^3^ and is recommended by several anaesthesiology and professional societies.^4^, ^5^, ^6^ However, despite these recommendations, adoption of this practice has been slow, and few clinicians and institutions currently implement this concept during high-risk surgery.^7^,^8^ One of the reasons is that proposed haemodynamic algorithms are complex and difficult to implement. Moreover, even when these protocols are applied by clinicians, their effectiveness remains limited as only 30% of fluid bolus initiated by anaesthesiologists resulted in appropriate increases in SV.^9^,^10^ This finding could be partly explained by the difficulty for the clinician to sustain attention throughout such high-risk procedures.

To overcome these limitations, a real-time clinical decision support system (The Acumen Assisted Fluid Management [AFM])  was incorporated on the latest version of the Hemosphere monitor (Edwards Lifesciences, Irvine, CA, USA) and was was designed to facilitate SV optimisation during high-risk surgery.^11^, ^12^, ^13^ This software uses machine learning to assess each patient's response to fluids and modifies fluid requirement thresholds to personalise fluid therapy. AFM is associated with decreased preload dependence,^11^ better predict fluid response rates after a fluid challenge,^10^,^13^ improved SV,^12^,^14^,^15^ optimised microcirculatory flow,^14^ and lower arterial lactate.^15^ Its effect on patient outcome, however, remains to be determined.

This international multicentre stepped-wedge cluster randomised trial involving high-risk patients undergoing high-risk abdominal surgery aims to demonstrate that implementing the AFM software (Edwards Lifesciences) can reduce postoperative complications when compared with routine care.

## Methods

The following article adheres to the Standard Protocol Items: Recommendations for Interventional Trials (SPIRIT) statement.^16^ The SPIRIT checklist is provided in Supplemental material 1. The trial sponsor is the Assistance Publique Hopitaux de Paris (APHP); Sabrina Williams is the head of the project at Paris (APHP) (Direction de la Recherche Clinique et de l’Innovation [DRCI] de l’AP-HP—Pôle Promotion, E-mail: recherche-innovation.aphp.fr).

### Trial design

**Figure 1:**
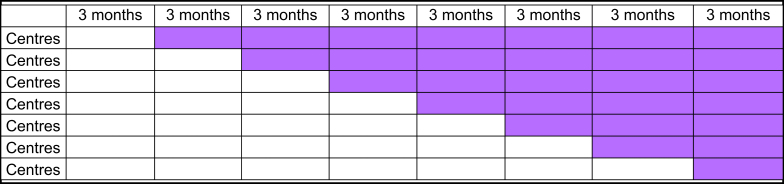
Stepped-wedge study design of the PEFLA trial. Considering the number of participating centres (16 centres) and the duration of this study (24 months), it was proposed to fix eight intervals of 3 months each. The randomisation will involve seven steps, each of them including two to three centres.

The PErsonalized FLuid Administration (PEFLA) trial is an investigator-initiated, international multicentre stepped-wedge cluster randomised controlled trial of 2000 high-risk patients undergoing high-risk abdominal surgery. A stepped-wedge design is a crossover design in which different clusters cross over (i.e. switch treatments) at different time points. The clusters typically cross over in one direction only, from control to intervention. The first time point usually corresponds to a baseline measurement where none of the clusters receive the intervention of interest (Fig 1). At baseline, all the patients will receive usual care. At subsequent time points, clusters will initiate the intervention of interest (i.e. AFM use), and the response to the intervention is measured. More than one cluster may start the intervention at a time point, but the time at which a cluster begins the intervention is randomised. All clusters eventually receive the intervention and the intervention is never removed once it has been implemented. Lastly, the stepped-wedge cluster design decreases the potential for a Hawthorne effect which is a type of reactivity in which individuals modify an aspect of their behaviour in response to their awareness of being observed.

### Study setting

This international multicentre trial is currently ongoing in 16 hospitals (12 French, two American, one Belgian, and one Canadian centre). Recruiting centres are hospitals having a high volume of high-risk abdominal surgery, as adapted from the European Society of Cardiology and European Society of Anaesthesiology noncardiac surgery risk score.^17^ It specifically includes major vascular surgery (abdominal aortic aneurysm repair and aorto bifemoral bypass surgery), duodeno-pancreatic surgery, liver resection, bile duct surgery, oesophagectomy, adrenal resection, total cystectomy, cancer debulking, hyperthermic intraperitoneal chemotherapy (HIPEC) surgery, and other types of complex and combined surgery.

### Participants and eligibility criteria

We will include consenting adult patients (aged 18 yr or older) scheduled for elective high-risk abdominal surgery (both open and laparoscopic/robotic assisted) who present at least one of the following comorbidities:

• American Society of Anesthesiologists physical status >2
• Classification exercise tolerance <4 metabolic equivalents as defined by the guidelines of the American College of Cardiology/American Heart Association
• Renal impairment (serum creatinine ≥1.3 mg dl^−1^ or >115 mmol L^−1^ or estimated glomerular filtration rate <90 ml min^−1^ (1.73 m^2^)^−1^ within the past 6 months) or renal replacement therapy
• History of coronary artery disease
• Chronic heart failure (New York Heart Association Functional Classification ≥II)
• Valvular heart disease
• Stroke
• Peripheral arterial occlusive disease
• Chronic obstructive pulmonary disease or pulmonary fibrosis
• Diabetes mellitus requiring oral hypoglycaemic agent or insulin
• Immunodeficiency because of a disease (e.g. HIV, leukaemia, multiple myeloma, solid organ cancer) or therapy (e.g. immunosuppressants, chemotherapy, radiation, steroids)
• Liver cirrhosis (any Child–Pugh class)
• Body mass index ≥30 kg/m^2^
• Current smoking or 15 pack-year history of smoking

We will not include patients who have cardiac arrhythmias (atrial fibrillation), who cannot give informed consent, who have no affiliation with the French health care system (for the centres in France only), who are participating in another interventional study that may impact the primary outcome, and who are pregnant.

Participants may decide to withdraw from the study at any time and for any reason. Investigators can temporarily or permanently withdraw a participant from the study for any safety reason or if it is in the participant's best interests. If a participant is lost to follow-up (i.e. cannot be located, contacted, or both), investigators will make every effort to reconnect with the participant, document their attempts in the source file, and determine if the patient is still alive. If a participant withdraws consent, any data collected before the date of premature exit may still be used.

### Study interventions

#### Control group: standard of care (as performed today in each centre)

Patients in the control group will receive the standard clinical practice of each centre's anaesthesia department regarding fluid therapy. The standard of care will consequently differ between centres. Some centres use routinely advanced haemodynamic monitoring devices to guide fluid administration, whereas others may not.

#### Intervention group: implementation of the Acumen assisted fluid management software

All patients in the interventional group will be monitored with the Hemosphere haemodynamic monitor (Edwards Lifesciences), which provides in addition to the usual advanced variables (SV, CO, SV variation, and systemic vascular resistance) fluid therapy decision support using the AFM software (Edwards Lifesciences). The AFM software (Edwards Lifesciences) has already been described in detail in previous studies.^13^ Briefly, the main functions of the software are to (1) integrate all monitored haemodynamic variables and continuously analyse a patient's fluid responsiveness; (2) analyse the haemodynamic response to fluid boluses; and (3) predict a patient's current fluid responsiveness and, when appropriate, prompt clinicians to consider a fluid bolus via the monitor's graphical user interface. To be able to do this, AFM uses SV, stroke volume variation (SVV), mean arterial pressure (MAP), heart rate (HR), and systemic vascular resistance (SVR) in two layers to predict fluid responsiveness. The first layer, or population model, uses data from previous populations to predict fluid responsiveness. The second layer, or bolus-log model, evaluates each patient's response to a fluid challenge. This layer will modify the thresholds of SV and other variables to those which are most highly correlated with an individual's fluid responsiveness. This tool thus determines when a patient is fluid responsive and modifies its targets to have the highest probability of predicting fluid responsiveness. In other words, it personalises fluid therapy. To increase compliance, physicians will have a training session before starting inclusion so that they are familiar with the use of AFM. To increase safety, anaesthesiologists will be able to discontinue and modify their haemodynamic strategies and therapies if deemed necessary.

More practically, all patients will have 2–4 ml kg^−1^ h^−1^ of baseline maintenance fluid therapy and additional fluid challenges (100–500 ml) based on the recommendations of the AFM system (Edwards Lifesciences). Ideally, each fluid challenge will be of 250 ml but anaesthesiologists can decide to give 100 ml or 500 ml depending on the clinical situation during the surgery.

#### Relevant concomitant interventions

All components of anaesthesia care will be left at the discretion of the attending anaesthesiologists and institutional guidelines in both groups other than haemodynamic management (fluid therapy) in the AFM group. Mean arterial pressure will be maintained >65 mm Hg during surgery in both groups.

### Primary and secondary outcomes

The primary outcome is a composite of all-cause mortality and major postoperative complications within 30 days after surgery defined as any of the following: acute myocardial injury (including myocardial infarction), acute kidney injury (all stages), severe infectious complications (including deep surgical site infection, pneumonia, sepsis, peritonitis), anastomotic leakage, pulmonary embolism or venous thrombosis, pulmonary oedema, acute respiratory distress syndrome, *de novo* arrhythmia, stroke, reoperation for any cause, non-fatal cardiac arrest, and mortality.

The outcome is met if any of these events occurred. Definitions of these complications are based on recent guideline papers on standard definitions of outcomes in perioperative medicine.^18^, ^19^, ^20^

Secondary outcomes consist of

• Incidence of each of the individual components of the composite primary outcome
• Incidence of the composite primary outcome within 7 days after surgery
• Incidence of a composite of postoperative infection rate within 30 days of surgery. This is defined as one or more of the following infections: surgical site infection, organ space surgical site infection, urinary tract infection, laboratory-confirmed bloodstream infection or infection, source uncertain (this is defined as an infection which could be more than one of the mentioned infections but it is unclear which)
• Clavien–Dindo classification and the modified Comprehensive Complication Index scores based on collected outcomes at postoperative day 30
• Hospital length of stay
• Incidence of unplanned hospital re-admission within 30 days after surgery
• Mortality at 90 days after surgery

An outcome adjudication committee will not be used in this trial. All study outcomes will be prospectively assessed by medical doctors or surgeons or intensive care physicians.

### Health economic analysis

We will also conduct a within-trial cost and cost-effectiveness analysis, focusing only on interventions directly evaluated in the trial from the hospital and payer's perspectives. This will be published later as an ancillary substudy of the original one.

### Sample size calculation

On the basis of data from previous published studies from our group, the percentage of patients with at least one major postoperative complication using a standard of care practice varies between 45% and 48.7%. As such, we decided to choose 46.5% as the incidence of postoperative complications in the control group. For a classical randomised trial, 466 patients per group (932 patients in total) are needed to show a 20% relative reduction in the primary outcome (from 45% to 36%), using a power of 80% and (two-tailed) alpha risk of 5%. Of note, the main assumption in randomised controlled trials is that the outcome of an individual patient is completely unrelated to that of any other patient (i.e. they are said to be ‘independent’). This assumption is violated in cluster randomised trials as patients within one cluster (centre in our case) are more likely to respond in a similar manner. A measure of this similarity is known as the intraclass correlation coefficient (ICC). Because of this lack of independence, larger sample sizes are required. The ICC values usually described in the literature and reported by the University of Aberdeen on a database dedicated to ICC (https://www.abdn.ac.uk/hsru/what-we-do/tools/index.php) range between 0.005 and 0.05.

The following criteria must consequently be considered when determining sample size for this study: the randomisation sequence (eight periods for each cluster/centre), time periods (3 months), average number of patients per centre (around 15 patients by period), and the coefficient of variation of cluster size, defined as the ratio of the standard deviation (SD) of cluster sizes. To estimate sample size by considering these additional criteria, Stata (StataCorp, College Station, TX, USA) routine stepped-wedge developed by Hemming and Girling has been used. In our trial, 1680 patients (*n*=840 patients by group) are needed to detect a difference between 46.5% and 36% for an ICC value at 0.01, a two-sided type I error at 5%, and a statistical power greater than 80%. To take into account a possible drop-out rate of at least 16% as 30-day follow-up may be difficult in this population, we will include 2000 patients in total. No planned interim analysis will be conducted during the study.

### Recruitment

Screening and recruitment will be performed during the preoperative anaesthesiology consultation. The screening visit takes place between 2 months and no later than the day before the inclusion visit (Table 1). As each centre has more than 200 patients who undergo a high-risk abdominal surgery each year, we expect 40% of the eligible patients to accept to participate in the study. The high recruitment is because of our study being considered as a quality improvement project where at one point the haemodynamic management is changed for a potentially better strategy (i.e. AFM-based goal-directed fluid therapy (GDFT)).

**Table 1:** Study timeline summary.

	Selection visit −2 months to day −1	Inclusion Day −15 to day −0	Intervention Day 0	Follow-up visit at day +30	Follow-up visit by phone at day +90
Verification of inclusion and exclusion criteria	X				
Informed consent	X				
Signature of consent	X				
Medical history or comorbidities		X			
Clinical examination		X			
Anaesthesiology consultation		X			
Intraoperative data			X		
Assessment of and morbidity and mortality				X	X
Assessment of mortality					X

### Randomisation

Randomisation will be performed using block randomisation with a sequence generated in Stata software (version 13; StataCorp) by an independent statistician. The randomisation will be stratified to ensure an alternation of low–medium-volume centres and high-volume centres. The randomisation list will be uploaded into the web-based CleanWeb® (Télémédecine, Boulogne-Billancourt, France) electronic case report form (eCRF) software and forwarded to the sponsor's quality assurance team for validation.

The eCRF (CleanWeb®) also provided clinicians with reminders about the current period (standard of care or AFM). Patients and investigators will not be blinded to treatment allocation. Centres will be notified of their randomisation status only 1 month before transitioning from the standard of care to AFM implementation. This time will be required to upgrade their existing haemodynamic monitoring devices with the AFM software (Edwards Lifesciences).

### Blinding and procedures to minimise bias

Because it is not possible to predict which operations will occur during a cluster's time period and all patients within that period receive the same intervention, selection bias is unlikely. Although it is not feasible to keep physicians blinded throughout the study, research personnel responsible for filling up/managing the eCRF will remain blinded to the intervention period, as they will not be informed about its timing.

### Data collection

Data will be recorded by research staff directly in the eCRF. The data will be collected in a centralised eCRF platform (CleanWeb, Télémédecine). An experienced data manager of the University Paris-Saclay Research Service will program this clinical data base and validate the data quality. Data will be collected from all included patients regardless of the allocated intervention or not.

### Statistical analysis

Analyses will be performed using Stata software (version 15, StataCorp). Baseline characteristics (centres and patients) will be presented as the mean and standard deviation or the median and interquartile range for each intervention group for continuous data and as the number of patients and associated percentages for categorical parameters. The results will be expressed using standardised differences and 95% confidence intervals (95% CIs). The characteristics of the patients and clusters will be summarised by intervention group to allow consideration for selection biases and lack of balance. Patients will be described and compared between intervention groups at baseline for eligibility and epidemiological, clinical, and treatment characteristics. Protocol deviations and reasons for withdrawal will be described. Other parameters such as the numbers analysed, the average cluster size, cluster characteristics, and important patient characteristics will be compared in each cluster by period. Analysis will be done according to the intention-to-treat principle.

To compare the incidence of the primary endpoint, a robust random effect Poisson generalised linear mixed model regression with robust variance will be proposed. Intervention groups, steps of randomisation, time periods—treated as categorical variables—and their interactions will be evaluated as fixed effects and centre and time as random effect. Results will be expressed as relative risks and 95% CIs. The estimated intra-cluster correlation and time effect from the fitted model will be reported. Multivariable analysis will use the same statistical model with covariates determined according to unbalanced baseline and to the clinical relevance (type of surgery, ASA score, comorbidities, and AFM compliance). Furthermore, interaction terms in the regression model will be used to test for heterogeneity of effect between subgroups: type of surgery, ASA score, comorbidities, AFM compliance, and anaesthesia duration. Between-group comparisons for other endpoints will be performed using same random effects models taking into account between- and within-centre and time variability (if necessary to obtain a normal distribution, a logarithmic transformation will be considered) or generalised linear. The time-to-event curves will be calculated with the Kaplan–Meier method and compared, when appropriate, using marginal Cox proportional hazards regression model taking into account between-and within-centre and time variabilities (90 days mortality). No missing data are expected for the primary endpoint. For the secondary endpoints, the statistical nature of missing data will be studied, and sensitivity analysis will be proposed to analyse the impact of missing data on results and propose the most appropriate method of imputation. More precisely, missing data mechanisms will be evaluated to determine if data are missing completely at random (MCAR), at random (MAR), or not at random (MNAR). Multiple imputation methods will be considered for handling MAR data, and sensitivity analyses (e.g. complete case analysis, worst-case imputation) will assess the robustness of results.

All analyses will be performed using Stata software (StataCorp). The widths of CIs will not be adjusted for multiplicity and cannot be used in place of hypothesis testing.

### Data monitoring

The sponsor is responsible for ensuring that all parties involved in the study provide direct access to all study locations, source data, source documents, and reports, to facilitate the sponsor's quality control and audit procedures, and inspections by the competent authority. The investigators will ensure that data monitoring personnel have access to the patient's documents and personal data strictly necessary for these tasks. All necessary steps will be done to maintain patient confidentiality.

## Ethics and dissemination

The study protocol (APHP220817) has been approved by a French ethics committee (Comité de protection des personnes Sud-Est II, Lyon) on 12 May 2023. All non-French participating centres have also full ethical approval. The study was also registered prospectively on ClinicalTrials.gov under the number NCT06011187 on 25 August 2023. Written informed consent will be obtained from all patients included in this study by investigators. The following article adheres to the SPIRIT statement (Supplementary material 1).

Upon completion of this work, a manuscript will be submitted for publication in a high-impact factor journal. We may also carry out *post hoc* analyses on study data. This work will also be presented at international scientific conferences. Sensible requests for data sharing will be considered by the study director only if they guarantee to preserve the confidentiality of the information requested. Authorship of manuscripts submitted for publication will follow the criteria defined by the International Committee of Medical Journal Editors. Authors' appearance will be based on the number of patients recruited at their centre, as predefined by the study director.

### Trial status

Study recruitment started on 1 February 2024. The estimated study completion date is 3 January 2026, and 30 April 2026, for the 90-day follow-up.

## Funding

French Ministry of Health (Programme Hospitalier de Recherche Clinique national, PHRC 2022); Edwards Lifesciences, Irvine, CA, USA (partial).

## Declarations of interest

SC, DV, PGG, AS, and PG received honoraria from Edwards Lifesciences for delivering lectures. BC received funding for an investigator-initiated research proposal, lecturing fees for presentations at industry-sponsored symposia and honoraria for participation to advisory boards from Edwards Lifesciences, lecturing fees for presentations at industry-sponsored symposia and honoraria for participation to advisory boards (Nordic Pharma), and lecturing fees for presentations at industry-sponsored symposia (AOP Health). PGG received fees for lectures from Edwards, Vygon, and AOP and is a consultant for Abbot. MC has ownership interest in Sironis, and Sironis has developed a closed-loop fluid system whose software is used in the Assisted Fluid Management System (Edwards Lifesciences, Irvine, CA, USA). The other authors declare that they have no conflicts of interest.
